# ‘Regrets become a lasting source of pain’: A qualitative study on family caregivers’ experiences leading up to a relative’s death

**DOI:** 10.1177/02692163251316677

**Published:** 2025-02-10

**Authors:** Hui-Ju Liang, Qian Xiong, Peng-Chan Lin, Jui-Hung Tsai, Nancy Preston

**Affiliations:** 1 Division of Health Research, Faculty of Health and Medicine, Lancaster University, Health Innovation One, Lancaster, UK; 2 Centre for Ageing Research, Division of Health Research, Faculty of Health and Medicine, Lancaster University, Lancaster, UK; 3 Department of Oncology, National Cheng Kung University Hospital, College of Medicine, National Cheng Kung University, Tainan, Taiwan; 4 Centre for Hospice Palliative Shared Care, National Cheng Kung University Hospital, College of Medicine, National Cheng Kung University, Tainan, Taiwan; 5 International Observatory on End of Life Care, Division of Health Research, Lancaster University, Lancaster, UK

**Keywords:** Death preparation, decision making, caregiving, bereavement, end-of-life care, family involvement, regret, Taiwan

## Abstract

**Background:**

Death preparations can benefit families both before and during bereavement. While these preparations are culturally influenced, evidence from non-Western cultures, like Eastern Asia, is limited.

**Aim:**

To explore how family caregivers prepare for a relative’s death in Taiwan.

**Design:**

A qualitative interview study analysed with reflexive thematic analysis.

**Setting/participants:**

Twenty-two primary family caregivers following a death involving specialist palliative care were interviewed.

**Results:**

An overarching theme was ‘getting everything right to have no regrets between the dead and the living’. Within this, two themes focussed upon improving the dying relative’s outcomes and the families’ subsequent bereavement: (1) ‘making the right end-of-life decisions is crucial but complex’, exploring preparations to involve (or not) the dying relative in making choices to minimise regrets. Participants often felt they understood the dying relative’s wishes so respected their preferences while maintaining family harmony through consensus-building and professional guidance. (2) ‘becoming a competent caregiver is the priority’, addressing preparations for fulfilling responsibilities, making sacrifices and developing caregiving competence to ensure the dying relative’s comfort. This would help reduce feelings of regret about not having done enough.

**Conclusion:**

Preparing for end-of-life decisions and caregiving is important for participants to reduce regret, benefiting subsequent bereavement. Of particular importance is family involvement and consensus-building in end-of-life decisions, reflecting Taiwan’s family-led culture. These findings can inform clinical practices in family-centric decision-making cultures where healthcare workers should be aware of the need to build consensus, sometimes without involving the dying person. Future research should include patients’ and healthcare professionals’ perspectives.

**What is already known about the topic?**

- Preparing families for a relative’s death in palliative and end-of-life care is important, involving medical, psychosocial, spiritual and practical tasks to enhance cognitive, behavioural and emotional readiness.
- Adequate death preparation benefits families’ experiences before death and during bereavement and is shaped by cultural contexts.
- Research on family caregivers’ death preparation primarily focusses on Western societies, overlooking insights from non-Western cultures like Eastern Asia, which could hinder culturally appropriate care.

**What this paper adds?**

- Preparing for ‘right’ end-of-life decisions and caregiving was important for participants’ death preparation, which aimed at reducing future regrets and benefiting subsequent bereavement.
- Protecting the dying relative through varying levels of involvement, respecting their preferences, maintaining family harmony through consensus-building and being supported by professionals’ expertise increased the potential for the best end-of-life decisions.
- Preparations to reduce regret about not having done enough involved fulfilling care responsibilities, developing caregiving competence to ensure the dying relative’s comfort, making sacrifices and fulfilling their last wishes.

**Implications for practice, theory or policy**

- The findings provide culturally appropriate guidance for preparations before death to help families feel they have minimised regrets, including assisting them in making the best possible end-of-life decisions, fulfilling care responsibilities, ensuring they feel they have done their best and preparing them to provide competent caregiving to ensure the dying relative’s comfort.
- Clinicians should discuss end-of-life care plans with family members, recognise the importance of building family consensus and recommend including the patient in these discussions where possible when caring for patients from family-centric cultures.
- Further research is needed to explore the role of autonomy in end-of-life decision-making and death preparation from patients’ and healthcare professionals’ perspectives.

## Introduction

Family preparation for death involves completing medical, psychosocial, spiritual and practical tasks^1,2^ for improving cognitive, behavioural and emotional readiness for the death,^3^ which shapes families’ end-of-life care experiences and subsequent bereavement.^4–6^ Adequate preparation alleviates fear of death, enables families to be present at the end of life and makes the impending death more acceptable while also easing the bereavement experience.^5–7^ Inadequate preparation is linked to poor bereavement adjustment,^4,8,9^ leading to complicated grief and distress, depression and anxiety.^3,10^ Feelings of regret or guilt of not doing enough for the deceased before death are common among bereaved individuals.^11–14^ However, existing evidence fails to identify which aspects of preparation influence bereavement.

Informal caregivers, often family members, are key in palliative and end-of-life care^15^ due to their increased responsibilities as death nears.^16,17^ They usually coordinate with healthcare professionals, make end-of-life decisions,^18^ and deliver direct care. Families become aware of the impending death through caregiving^19^ to meet complex care demands and prepare for death.^20^ Most research on family caregivers’ death preparation is conducted in Western societies,^5–7,20–25^ limiting its cross-culture transferability.^26^ Research from non-Western world, such as Eastern Asia,^27–31^ is limited. Such studies often focus on identifying the relationship between death preparation and caregiving burden using a quantitative approach with standardised measurements.^29^ For example, a survey in Taiwan showed that 60% of family caregivers felt insufficiently prepared for a relative’s death,^30^ but it offers limited insights into why people have these experiences and the key components of preparation.

Taiwan’s palliative care services are funded through National Health Insurance and are well-developed to cover people with cancer and non-cancer diagnoses.^32^ Ensuring patients’ autonomy during advance care planning consultations is a legal responsibility stipulated by The Patient Right to Autonomy Act (2016).^33^ Advance care planning in the West enhances family caregivers’ death preparedness,^7,21,34^ understanding of end-of-life processes,^21^ and coping with impending death.^34^ Taiwan’s palliative care framework offers a valuable context for investigating families’ death preparation. This paper draws upon data collected for a PhD project^35^ and focusses on family caregivers’ experiences of death preparation before their relative dies and how the preparation affects subsequent bereavement using qualitative methods.

## Methods

### Design

A qualitative interview study was conducted, followed by Braun and Clarke’s reflexive thematic analysis^36^ that allows for exploring experiences to develop meaningful patterns of death preparations for generating a better understanding in a broad context.^36,37^ It is underpinned by critical realism acknowledging that good experiences of death preparations exist but are contextually influenced.^36,38^ Reflexive Thematic Analysis Reporting Guidelines ensured study quality and thorough reporting.^36,39–41^

### Setting

The study aimed at families whose relatives had experienced specialist palliative care in Taiwan. Taiwan’s specialist palliative care services include multidisciplinary teams with advanced training, offering hospice inpatient, consultation and home care services.

### Participants

Participants were family members who were primary caregivers of a relative who received specialist palliative care before death, aged 20 years or older (recognised as an adult in Taiwan before January 2023^42^), fluent in Mandarin or Taiwanese. They had bereaved 6–18 months,^19,23^ an appropriate timeframe allowing for grieving^43^ and recall of experiences. Individuals bereaved for deaths of children (under 20 years old) were excluded due to significant differences from adult deaths.^44^

### Sampling and recruitment

Purposive and snowball sampling methods were employed for recruitment. Specialist palliative care teams holding records of deceased patients for bereavement support were initially contacted to help recruit potential participants. The consent process encouraged questions and discussion, and written informed consent was obtained before all interviews.

### Data collection

Following a pilot interview in September 2022, H-JL conducted semi-structured interviews with a literature-informed topic guide (Supplemental Appendices 1)^5,19,21,23,24^ from October 2022 to March 2023, either in-person at participants’ convenient places or online complying with the COVID-19 policies. Demographic data were collected (Supplemental Appendices 2). Interviews were audio-recorded and had flexible durations. A distress protocol was developed^45^ to ensure safety (Supplemental Appendices 3).

H-JL’s background as a palliative care nurse might affect interviews and data interpretation. Thus, she kept a reflexive journal to challenge assumptions in real-time and reflect on personal feelings and thoughts.^36^

Recruitment was concluded based on information power that continually assessed data richness to ensure the research questions were sufficiently addressed.^36,46^

### Ethical approval

Ethics approval was received from the Hospital (A-ER-111-193) and Lancaster University (FHM-2022-0972-ExRev-1) Research Ethics Committees.

### Data analysis

H-JL conducted data analysis recursively following the six phases of reflexive thematic analysis: familiarising with the dataset, coding, generating initial themes, developing and refining themes and writing up.^36^ Interview recordings were transcribed in Traditional Chinese and managed with NVivo. Coding was conducted in Traditional Chinese for close engagement with the data and to improve analysis quality.^47^ Selected code labels and relevant data were translated into English for team discussions, including NP being an English speaker, to enhance understanding, interpretation of the data and researcher reflexivity throughout the analysis process.^36^

H-JL critically engaged with the dataset, conducted two rounds of inductive coding and translated final codes into English with certain Traditional Chinese terms retained. H-JL then generated, reviewed and refined themes by clustering related codes, creating thematic maps (Supplemental Appendices 4), defining each theme and developing a coherent narrative.

## Findings

### Participants characteristics

Twenty-two primary family caregivers from seven hospitals with specialist palliative care units were interviewed (Table 1). The hospitals included four medical centres, two regional hospitals and one district hospital across Southern (*n* = 3), Central (*n* = 2), Northern (*n* = 1) and Eastern (*n* = 1) Taiwan. Participants’ mean age was 55.3 years (range = 23–78 years), including 8 men and 14 women, mainly adult children of people who died, employed full-time and followed Taiwanese folk religion. The average bereavement period was 10.7 months (range = 6–17 months). The 21 deceased patients, averaging 70.9 years old (range 44–93 years), were mostly women and mainly diagnosed with cancer. Nearly half died at home, while the rest were in hospice or non-hospice inpatient units. Interviews averaged 115 min (range = 70–186 min).

**Table 1.** Descriptive characteristics of family members and deceased patients.

Bereaved family members (*N* = 22)		Deceased patients (*N* = 21)	
Age		Age at death	
20–40	2	41–65	7
41–65	15	66–80	9
66+	5	81+	5
Gender		Gender	
Man	8	Man	4
Woman	14	Woman	17
Employment status		Primary medical diagnosis	
Full time	12	Cancer	14
Retired	8	Non-cancer	7
Unemployed	2	Specialist palliative care received before death	
Religious beliefs		Inpatient, consultation and home care	7
Taiwanese folk religion	8	Inpatient and consultation care	4
Buddhism	3	Inpatient and home care	3
Taoism/Daoism or Yiguandao	3	Inpatient care	2
Christianity/Catholic	6	Home care	5
No affiliation	2	Death of place	
Relationship with deceased patients		Home	10
Adult children	14	Hospice inpatient unit	9
Spouse	5	Non–hospice inpatient unit	2
Sibling	2		
Parents of adult child	1		
Bereaved time before recruitment (months)			
6–12	18		
13–18	4		

### Themes

An overarching theme of ‘getting everything right – to have no regrets between the dead and the living’ was developed as a vital goal in death preparation. Participants aimed at doing their best in death preparation to minimise future regrets, reduce the dying relative’s regrets and ensure their comfort:

*It’s necessary to ensure that the person who’s about to leave [referring to dying] has no regrets but also making sure that those close to them don’t carry regrets in their hearts. (Daughter, FC4)*

*‘Having no regrets between both sides [referring to the dying relative and families]’ (Daughter, FC18)* was emphasised. Participants noted that *‘regrets become a lasting source of pain’ (Father, FC13)* and that *‘there will be no chance to do it again’ (Daughter, FC4)*, leading to *‘self-blame’ (Daughter, FC18)* and *‘guilt’ (Daughter, FC3)*, which complicated bereavement. As described, *‘To have less grief means not feeling regrets’ (Father, FC13)*. While phrases like *‘having no regrets’ (Daughter, FC18)* and *‘not feeling regrets’ (Father, FC13)* may seem unrealistic, participants used them to stress the need to reduce regrets when preparing for a relative’s death, recognising their impact on bereavement.

**Figure 1.**
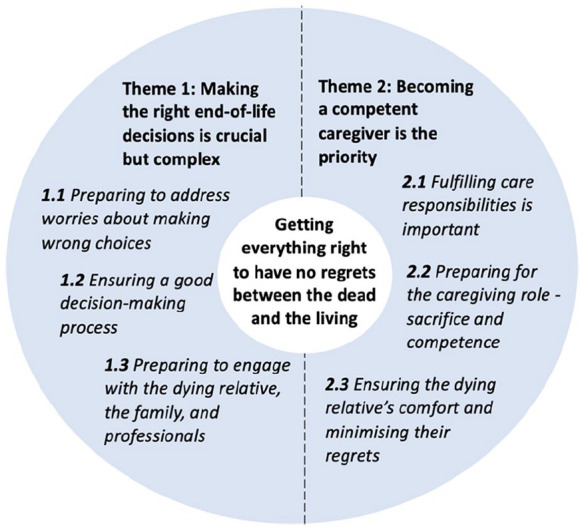
Families’ preparations and actions before the relative’s death.

This overarching theme was broken down into two themes focussed upon improving the dying relative’s well-being leading up to the death: (1) ‘making the right end-of-life decisions is crucial but complex’ and (2) ‘becoming a competent caregiver is the priority’. These themes highlight families’ preparations and actions before the death, specifically regarding regrets and their impacts on subsequent bereavement (Figure 1).

#### Theme 1: Making the right end-of-life decisions is crucial but complex

This theme explores the complexity and importance of preparing for the ‘right’ end-of-life decisions, including how and why families make these choices and what they mean to them.

##### Preparing to address worries about making wrong choices

Preparing to address worries about making wrong choices as death approached was emphasised. Participants expressed anxieties such as *‘Did I make any wrong decisions?’ (Daughter, FC10)* and *‘I worried that this choice for my mother was wrong’ (Daughter, FC12)*. They were afraid that decisions could lead to *‘increased suffering’ (Husband, FC19)*, *‘unnecessary treatments’ (Daughter, FC17)* or shorten the relative’s life, causing *‘unforgivable guilt’ (Father, FC13*). These concerns often revolved around decisions regarding withholding or withdrawing life-prolonging treatments like *‘removing a nasogastric tube’ (Daughter, FC12)* and ‘*Do-Not-Resuscitate’ (Daughter, FC9)*:

*To be honest, when faced with signing a Do Not Resuscitate form, I really couldn’t bring myself to do it. Overcoming that barrier was extremely challenging. It was very, very difficult. That feeling was truly frightening. I mean, once I signed, it felt like my mother was going to pass away soon. (Daughter, FC17)*

Participants addressed these concerns by striving to get choices ‘right’, making the best possible end-of-life decisions through a good decision-making process and involving all essential parties, as detailed below.

##### Ensuring a good decision-making process

Preparing for a good decision-making process was part of making the best possible end-of-life decisions. This included easing the dying relative’s burdens and shielding them from negative emotions like ‘*fear’ (Sister, FC5)*. As expressed, ‘*We didn’t want to make my mum feel like her life was coming to an end*’ *(Daughter, FC7)*. Participants thus found it essential to determine whether, when and how to involve the dying relative, leading to varying inclusion levels. Exclusion happened if the dying relative showed disinterest or if decisions were guided by families’ knowledge about them and topic sensitivity. Selective involvement aimed to protect the dying relative, while inclusion was necessary if they preferred to decide independently, highlighting the importance of aligning the process with their preferences:

*In the past, my mum always made decisions for herself. So, I also hoped that in the final moments of her life, she could follow her own wishes and make her own decisions. (Daughter, FC18)*

Protecting the dying relative during decision-making also influenced how distressing information was disclosed. Participants felt *‘guilt’ (Daughter, FC2)* if inappropriate disclosure caused suffering. Concerns included *‘potential impacts on the illness condition’ (Husband, FC20)*, *‘collapse’ (Daughter, FC2)*, emotional distress like *‘shocking, overwhelming’ (Daughter, FC9)* or *‘disrupted joy in life’ (Husband, FC19)*. This information included medical details like *‘life expectancy’ (Daughter, FC9)* and non-medical issues such as other family members dying or becoming unwell. Consequently, participants often withheld full disclosure or provided only vague details:

*I asked the physician how much time my young sister had left, and he said approximately three months. However, I only told her that her treatment was no longer effective and did not reveal the specific time remaining. [The patient did not ask about her lifespan] (Sister, FC5)*

##### Preparing to engage with the dying relative, the family and professionals

Preparing to engage with the dying relative, family and professionals was important to make the best possible end-of-life decisions. While direct involvement of the dying relative was not always essential, respecting their preferences was vital. As expressed, *‘It’s so important to respect my mother’s preferences’ (Daughter, FC18)*. Therefore, planning to understand the dying relative’s end-of-life preferences through discussions like *‘advance care planning consultation’ (Son, FC16)* was necessary. Preferences varied from general principles like *‘don’t make me suffer’ (Daughter, FC17)* to specific ones like ‘*Do Not Resuscitate’ (Daughter, FC9)*. Without explicit instructions, participants needed to infer potential preferences based on their understanding of the dying relative:

*Knowing my husband’s personality, I was aware that he wouldn’t want to continue lying in bed and be cared for by others. So, I made the decision about removing a nasogastric tube for him. (Wife, FC14)*

Preparing to *‘maintain family harmony’ (Daughter, FC18)* by reaching consensus in end-of-life decision-making was essential. As expressed, *‘I feared my sisters might oppose our mother’s advance directive and see it as me acting unilaterally’ (Son, FC16) ‘caregiving matters’ (Daughter, FC18)*. This emphasised the need for family consensus on decisions, including, to avoid conflicts:

*If one of my siblings didn’t agree with the decisions I made for my mother, the outcome couldn’t be satisfied like it is now. So, I believe that it’s important to have a consensus among the family. (Daughter, FC17)*

Preparing to engage with healthcare professionals was important for making the best possible end-of-life decisions. Participants, often *‘non-professionals’ (Daughter, FC3)*, found it challenging to act as intermediaries in *‘the professional decision-making process’ (Daughter, FC12)*. Gaining knowledge in advance through ways like ‘*searching for information’ (Sister, FC5)* improved their communication with professionals. As expressed, ‘*I would know how to ask medical staff questions after doing some research’ (Daughter, FC7)*. Additionally, obtaining professionals’ expertise and medical information about the dying relative through *‘family meetings’ (Wife, FC1)* was essential in enhancing participants’ confidence and moving them beyond relying solely on personal opinions:

*The family meeting process gave me the feeling that the decision [referring to removing a nasogastric tube] we made for my mother was reviewed and supported by the medical team, not just made on our own, but I had a team to support it. (Daughter, FC12)*

Preparing for the ‘right’ (the best possible) end-of-life decisions to minimise regrets included ensuring a process that shielded the dying relative from stress, aligned with their preferences, maintained family harmony through consensus-building and relied on professionals’ expertise.

#### Theme 2: Becoming a competent caregiver is the priority

Why and how family members prepare to become competent caregivers and actions taken to maximise the dying relative’s comfort, the primary goal of death preparation, are explored in this theme.

##### Fulfilling care responsibilities is important

Fulfilling care responsibilities before the death helped minimise regrets afterwards. Familial duty and affection motivated participants to care for their dying relatives. As expressed, *‘I had to fulfil the responsibility as a husband’ (Husband, FC19)*. Adult children saw *‘caring for sick parents’ (Son, FC22)* as a way to honour *‘filial piety’ (Daughter, FC18)* and repay their nurturing, regardless of past relationship quality. For instance, *‘Caring for my mother was a way of paying her back for raising me’ (Son, FC22)*. This approach helped avoid the regret of ‘*wanting to care for parents, but they are no longer around’ (referring to their death) (子欲養而親不在, Zi yù yǎng ér qīn bùzài) (Daughter, FC4)*. Showing gratitude for the dying relative’s past contributions to the family through caregiving was also important:

*My wife hasn’t experienced much happiness since we got married. We’ve faced challenges from the beginning, and she’s stood by me through it all. That’s why I’ve made it a priority to accompany and care for her to the best of my ability. (Husband, FC15)*

Fulfilling caregiving responsibilities to minimise regret was perceived as essential, necessitating thorough preparation for this role, as described below.

##### Preparing for the caregiving role – Sacrifice and competence

Preparing for caregiving involved making sacrifices and developing competence. These sacrifices prioritised the dying relative’s needs over their own and gave participants a sense of having *‘fulfilled the utmost responsibility and done everything possible’ (Husband, FC19)* and helped *‘minimise regrets’ (Daughter, FC12)* after death. Sacrifices included adjusting personal lives to *‘move back to live with my mother to care for her’ (Daughter, 18)* and *‘focus almost entirely on my young sister apart from work’ (Sister, FC5)*, as well as neglecting personal physical, social and emotional needs at times, such as *‘enduring a painful knee from frequently climbing stairs while caregiving’ (Husband, FC19)*, ‘giving up *all volunteering activities’ (Daughter, FC18)* and *‘making a lot of efforts to maintain emotional stability while caring for my mother’ (Daughter, FC12)*.

Witnessing the dying relative’s suffering was described as *‘unbearable’ (Son, FC11)*, causing mental pain like *‘helplessness, self-blame’ (Daughter, FC3)*, *‘guilt’ (Daughter, FC4)*, feeling *‘heartbroken’ (Daughter, FC6)* and ‘*deep pain in heart’ (Husband, FC19)*. Participants feared they could not ease or might worsen the suffering through inadequate caregiving. Despite this, caregiving also evoked positive feelings of being *‘relieved’* and *‘more at ease’ (Sister, FC5)*. Participants developed their caregiving competence by *‘acquiring a lot of knowledge’ (Son, FC22)* and learning new skills through *‘guidance from healthcare professionals’ (Husband, FC15)*, *‘training courses’ (Son, FC22)* and *‘online resources’ (Sister, FC5)*.

##### Ensuring the dying relative’s comfort and minimising their regrets

Preparations to ensure the dying relative’s comfort and reduce regrets before death included competent caregiving and selecting an appropriate care location. Preferences varied between hospitals with *‘palliative care inpatient units’ (Wife, FC1)*, valued for their *‘medical staff’s expertise’ (Husband, FC15)* and *‘assisted bathing equipment’ (Daughter, FC10)* and care at home, which offered ‘*a sense of security’ (安全感, ān quán gǎn) (Daughter, FC17)* in a familiar environment. Home care required preparing for symptom management like learning to administer *‘subcutaneous morphine’ (Husband, FC15)* and adjusting the home environment to meet the relative’s needs, including space and necessary medical devices:

*My brother’s room was transformed into my father’s room. We moved some furniture out and put a medical bed into the room. (Daughter, FC4)*

Preparing to provide personal care, including *‘changing nappies, adjusting positions’ (Daughter, FC12)* and *‘preparing meals’ (Daughter, FC4*), was essential for the dying relative’s cleanliness and comfort. Participants viewed food preparation as a way of *‘expressing care’ (Daughter, FC2)* and regarded food as vital for physical strength. They worried about their dying relative *‘being hungry’ (Son, FC11)* if they did not eat enough, motivating them to prepare meals to *‘supplement nutrition’ (Daughter, FC7)*. Therefore, preparing to understand and accept the dying relative’s reduced appetite and decreased need for food through *‘past experiences, reading materials’ (Daughter, FC2)*, and *‘professional explanations’ (Son, FC11)* was necessary.

Ensuring the dying relative’s ‘*happiness’ (Wife, FC1)*, *‘feeling loved’ (Husband, FC19)* and *‘inner peace’ (Daughter, FC12)* as they *‘approached the last moment of life’ (Wife, FC1)* was important. Preparation involved helping them *‘feel happy and joyful until the end’ (Husband, FC19)* by safeguarding their decision-making process, as discussed in Theme 1. This also included providing family companionship through *‘being surrounded by family members’ (Father, FC13)* and respecting their preferences for enjoyable activities, like *‘not making my husband do things he didn’t want to do’ (Wife, FC1)*. Adjusting interactions, including ‘*greeting and talking to my wife even when she could no longer communicate verbally’ (Husband, FC19)*, was essential for showing love. Additionally, preparing religious practices based on shared beliefs, such as *‘singing hymns’ (Daughter, FC12)*, *‘discussing religious texts’ (Son, FC21)* and performing rituals like *‘lighting a lantern’ (a practice from Yiguandao, which blends teachings from Confucianism, Taoism, Buddhism, Christianity and Islam) (Son, FC22)*, enhanced the dying relative’s inner peace and mental strength.

Preparing the dying relative to minimise regrets before death was vital. As expressed, *‘I told my husband, I didn’t want you to leave (referring to die) with regrets’ (Wife, FC1)*. This included help them review their life, asking questions like *‘Have you been happy in your entire life’ (Daughter, FC18*), and creating opportunities for *‘reconciliation’ (Son, FC11)* and heartfelt expressions between the dying relative and family members. Actions included *‘showing gratitude and saying sorry’ (Daughter, FC10)*, ‘*offering comforting words’ (Daughter, FC3*) and *‘resolving past conflicts’ (Daughter, FC4)*. Understanding and fulfilling the dying relative’s unfinished business or wishes was also important:

*About a month before my younger sister passed away, she was in the hospital and expressed her wish to see our father’s spirit tablet [a tablet with the deceased’s name, symbolising the soul of the deceased] and worship him. We sought permission from the hospital and brought her home to fulfil her wish. (Sister, FC5)*

Preparing to be competent caregivers was the priority, aiming to minimise future regrets by fulfilling caregiving responsibilities, making sacrifices and ensuring the dying relative’s comfort.

## Discussion

### Main findings of the study

Preparing for the ‘right’ end-of-life decisions and caregiving was important for Taiwanese families in the study during death preparation, aimed at minimising future regrets and benefiting subsequent bereavement. The best possible decisions included protecting the dying relative by deciding whether to involve them in discussions, maintaining family harmony through consensus-building and relying on professional expertise. Preparations to reduce regret about not doing enough involved fulfilling care responsibilities, making sacrifices, developing caregiving competence to ensure the dying relative’s comfort and addressing their last wishes to minimise regrets.

### What this study adds?

The study demonstrates how the cultural context shapes families’ end-of-life decision-making and caregiving for the dying relative during death preparation. Participants’ end-of-life decision-making reflects Taiwan’s family-led culture, which may conflict with the Western-centric model underpinning Taiwan’s palliative care practices. The time participants spent caring for a dying relative aligns with findings from Western studies,^20,24^ but our study reveals the deeper cultural significance of caregiving in Taiwan.

This study highlights that it was sometimes necessary to shield patients from direct participation in end-of-life discussions, with their level of involvement determined by families who aimed to ensure the best possible decisions. Patients were excluded if they showed no interest or based on families’ prior knowledge of the patients and the sensitivity of topics. These complex interactions between patients and families shaped their awareness of the impending death over time^48^ and influenced death preparation.^49^ However, the families’ exclusionary approach may conflict with Western perspectives on patient autonomy, prioritising individualism and opposing paternalism,^50^ and these views often influence medical practices in Taiwan.^51^ Instead, relational autonomy, which respects individual choice within social and cultural contexts, is more suitable for end-of-life situations across cultures,^50^ including family-centric societies.^52^ Our findings show families played a key role in decision-making^53^ that is viewed as a collective family responsibility in Taiwanese culture.^52^ In Taiwan, the Natural Death Act (2000)^54^ and the Patient Right to Autonomy Act (2016)^33^ mandate family participation in medical decisions, including their right to sign Do-Not-Resuscitate forms considering patients’ preferences^54^ and involving at least one family member in advance care planning.^33^ These laws acknowledge family roles in medical decision-making but may struggle to address the need for building family consensus in end-of-life decisions, aiming to avoid conflicts and maintain family harmony, as this study highlights. This reflects the central Taiwanese cultural emphasis on Confucian values of family-centredness^52,55^ and harmonious relationships.^55^ From the perspective of relational autonomy, patients’ preferences should take precedence over contextual factors, including family wishes, when they conflict.^50^ Thus, this approach is less applicable in the Taiwanese context, where family consensus is important despite potential misalignment with patients’ end-of-life care preferences.^56^ Our findings provide new insights into relational autonomy in end-of-life decision-making from the family perspective. Future research should investigate this concept from the viewpoints of patients and professionals.

Align with previous research, this study finds that families’ death preparation impacted their bereavement.^4,5,8^ Our study further highlights that getting end-of-life decision-making and caregiving ‘right’ was perceived as beneficial for subsequent bereavement by minimising regret. Regret, involving self-appraisal of past behaviours, often leads to painful emotions such as guilt, self-blame and a wish to change those actions if dissatisfied.^57,58^ Regret or guilt about not doing enough for the deceased before death is common among the bereaved,^11–14^ and such feelings can intensify grief, making bereavement more challenging.^59–61^ While bereavement theories typically focus on post-death experiences,^62,63^ our study offers preventative insights into reducing regrets beforehand to ease bereavement. However, this approach may not apply to sudden or unexpected deaths.

End-of-life decision regrets among families are underexplored,^64^ with limited evidence on their extent and contributing factors.^65^ For instance, a Taiwanese study found substantial decision regrets among family caregivers before and after the death,^66^ while a U.S. study linked life-prolonging treatment to increased regrets among African American families.^67^ These regrets likely stem from the complex decision-making process involving dying patients, families and healthcare professionals, as underscored in our study. Attempting to make the best possible end-of-life decisions was seen as beneficial in this study by mitigating regret from perceived wrong choices. This involved protecting the dying relative from too close involvement at times, maintaining family harmony and relying on professional expertise to avoid unnecessary suffering and treatments.

Evidence on the impact of end-of-life caregiving on bereavement is mixed.^9,61,68^ For example, a U.S. study found that positive caregiving aspects, such as feeling useful, were linked to higher grief levels.^68^ However, our study shows that being useful through fulfilling care responsibilities,^24,69^ making sacrifices,^70^ and ensuring the dying relative’s comfort^70,71^ were perceived as beneficial for bereavement. Families in our study also experienced emotional distress from witnessing suffering and feeling unable to help, leading to self-blame and guilt,^72^ complicating the bereavement. Our findings underscore self-sacrifice being important in caregiving, shaped by Taiwan’s collectivist culture prioritising others over individuals^73^ and Confucius values emphasising filial piety^55,74^ and the fulfilment of family care duties.^74,75^ This cultural significance of caregiving may help explain the positive impact of end-of-life caregiving on bereavement among Taiwanese families.^70^

Healthcare professionals were pivotal in families’ death preparation, including end-of-life decision-making and caregiving.^1,2,21,76^ However, families’ needs often remained unmet, even in palliative care settings.^77^ Future research should explore healthcare professionals’ perspectives on strategies for supporting family preparation. Suggestions for preventing challenging bereavement before death include assisting families in making the best possible decisions, fulfilling care responsibilities, ensuring they feel they have done their best and preparing them to provide competent caregiving to ensure the dying relative’s comfort. Additionally, clinical practices should prioritise family involvement and consensus-building in end-of-life decision-making through family meetings,^78^ in contexts where family-centric approaches are valued or when caring for patients and families from other cultural backgrounds in Western societies. Patients’ wishes and families’ knowledge about the patient can help clinicians decide whether to exclude the patient from end-of-life care discussions. When the patient is excluded, it is vital to guide families in respecting the patients’ expressed preferences or inferring potential preferences based on their understanding of the patient, especially when no explicit instructions have been provided.

### Reflections, strengths and limitations of the study

Families’ death preparation is contextual, including interactions with the dying relative, other family members, healthcare professionals, healthcare system and the broader social and cultural context. The main researcher (H-JL), a senior Taiwanese palliative care nurse, acted as a clinician-researcher, which might have influenced data generation and analysis^79^ due to her personal cultural background and preconceived views on death preparation. However, researcher reflexivity was fully considered.^36,79^ H-JL maintained a reflexive journal and had ongoing discussions with NP and QX to receive critical feedback, enhancing data interpretation and reflexivity.^36^ For example, the interviews did not explore participants’ views on what constituted ‘right’, ‘good’ or ‘best’ end-of-life decisions. Instead, H-JL reflected this point on the Taiwanese collectivist values, emphasising self-improvement through self-criticism.^73^ In Taiwanese society, criticism of poor decision-making is common, which may explain the focus on making ‘right’ decisions. In this study, ‘right’ was interpreted as ‘the best possible’.

The study’s strength is the diverse participant residences across Taiwan, achieved through purposive sampling, which enriched the data, allowed for a thorough exploration of research questions and enhanced its transferability to countries with family-centred decision-making.^36^ To preserve cultural meaning, findings were translated from Traditional Chinese to English later in the analysis, retaining certain Traditional Chinese words.^47^ Additionally, H-JL and QX, who spoke Chinese and English, facilitated exploring language nuances in both languages.

The study has some limitations, including potential issues with transferability to non-specialist palliative care settings and non-primary family caregivers. The predominance of adult children of deceased patients, mainly women, and the focus on people with cancer could also affect transferability.^80^ While we believe that participants’ experience of the entire death preparation process provided valuable insights, we acknowledge that the 6- to 18-month bereavement timeframe might have influenced the study’s findings, particularly the emphasis on preparation to reduce regret about not doing enough, a sentiment commonly felt by bereaved individuals.^11^ Future research should explore this topic in non-specialist palliative care contexts, focus on the period before the patient’s death, examine a wider range of relationships with the deceased, address various diagnoses and incorporate patients’ perspectives. Additionally, although both in-person and virtual interviews might affect data quality, previous research shows they yield similar rapport and depth of information,^81^ consistent with our study’s comparable interview durations.

## Conclusion

Preparing for end-of-life decisions and caregiving is important for Taiwanese families in the study to minimise regrets, benefiting subsequent bereavement. Family involvement and consensus-building in these decisions are essential in Taiwan, reflecting its family-led culture. These findings can inform clinical practices in family-centric decision-making contexts. Suggestions for preventing difficult bereavement before death include fulfilling caregiving responsibilities, providing competent care and relieving the dying relative’s suffering. Future research should include the perspectives of patients and healthcare professionals.

## Supplemental Material

sj-docx-1-pmj-10.1177_02692163251316677 – Supplemental material for ‘Regrets become a lasting source of pain’: A qualitative study on family caregivers’ experiences leading up to a relative’s death Supplemental material, sj-docx-1-pmj-10.1177_02692163251316677 for ‘Regrets become a lasting source of pain’: A qualitative study on family caregivers’ experiences leading up to a relative’s death by Hui-Ju Liang, Qian Xiong, Peng-Chan Lin, Jui-Hung Tsai and Nancy Preston in Palliative Medicine
