# Supplementary material for: ‘Regrets become a lasting source of pain’: A qualitative study on family caregivers’ experiences leading up to a relative’s death
Source: Palliat Med. 2025 Feb 10;39(3):401–12. doi: 10.1177/02692163251316677 (PMC11877984; doi:10.1177/02692163251316677)

**Supplementary File**

**Supplemental appendices 1:** Family Caregivers Interview Topic Guide

**Supplemental appendices 2:** Demographic Characteristics for Family Caregivers

**Supplemental appendices 3:** Interview Distress Protocol

**Supplemental appendices 4:** Thematic maps

**Supplemental appendices 1: Family Caregivers Interview Topic Guide**

- The research question of this interview study:

What are the experiences of preparedness for death and bereavement of family caregivers of terminally ill patients receiving specialist palliative care?

- The interview topics will cover:
- The triggers for conversations about preparing for death and bereavement.
- The components of preparedness for death and bereavement.
- The barriers and facilitators of preparedness for death and bereavement.
- The components of preparedness for death and bereavement benefiting for or worsening bereavement adjustment.

**NOTE:** Question order and wording will be tailored to the circumstances of participants.

1. **Introduction**

- Introduce the researcher self.
- Conversational dialogue to aid developing trust and rapport.
- Completion demographic characteristics related to family caregiver (Appendix 4); fill in by the researcher; demographic characteristics about the deceased will be completed during the interview.

1. **Background**
2. Information about the deceased

- Example: *Please tell me a little about his/her disease.*

1. Information about the family caregiver

- Example: *Please share about your experience of caring for him/her.*

1. **Experience of preparing the death of the relative for the family caregiver**
2. Initial awareness

- Example: *When did/how did you first realised that he/she might be dying（不久人世）or his/her disease status was worse*（病況不好/狀況不好）*?*

1. Tasks completed

- Example: *What tasks did you complete to prepare his/her impending death?*

1. Emotions experienced

- Example: *What were your emotions during this process?*

1. Challenges and resources

- Example: *What challenges did you encounter during this process?*

1. **Professional care offered for helping the family caregiver to prepare the death**

- Example: *What did health care professionals offer in terms of helping you to prepare for his/her impending death?*

1. **Bereavement experience of the family caregiver**

- Example: *Please share about your life during the time of his/her absence.*
- Example: *What effect did the experience of preparing the death have for your bereavement adjustment?*

1. **Conclusion**

- Example: *Is there anything else you would like to say or expand on?*
- Thanks for the participant.

**Supplemental appendices 2: Demographic Characteristics for Family Caregivers**

**NOTE:** This form will be completed by the researcher during the interview.

| Name: |
| --- |
| Gender  Male  Female  Prefer not to say |
| Age (years): |
| Employment status  Employed  Unemployed (currently not working)  Retired  Other |
| Relationship to Deceased  Spouse Children Grandchildren Parents Sibling Other (__________) |
| Information about Care Recipient |
| Age at Death: |
| Gender  Male  Female |
| Diagnosis  Cancer (____________________)  Non-cancer (____________________) |
| Time since your family member passed away: |

**Supplemental appendices 3: Interview Distress Protocol**

**Step 1: Aware of indications of distress during the interview**

Participants are experiencing a high level of emotional distress or are exhibiting behaviours that show the interview is too stressful such as uncontrolled crying, incoherent speech, and indications of flashbacks.

**Step 2: Immediate response**

- Offer to stop the interview.
- Offer support, allow the participant time to fully express emotions and regroup.
- Assess mental status:

1. Tell me what thoughts you are having.
2. Tell me what you are feeling right now.
3. Do you feel you are able to go on about your day?

- Determine if the participant is experiencing acute emotional distress beyond what would be normal expected in an interview about bereavement experience.

**Step 3: Taking actions based on situation of the participant**

- Continue the interview if the participant feels that he/she can carry on.
- Stop the interview completely if the participant feels that he/she is unable to continue. The researcher accompanies him or her until he/she feels okay and contact his/her family member or mental health provider if needed.

**Step 4: Follow up**

- Offer helpful information about available support and resources if needed.
- Encourage the participant to seek professional help (e.g., mental health provider).
- The researcher will contact the participant the next day to see if he/she is okay, with his/her permission.

**Supplemental appendices 4: Thematic map**


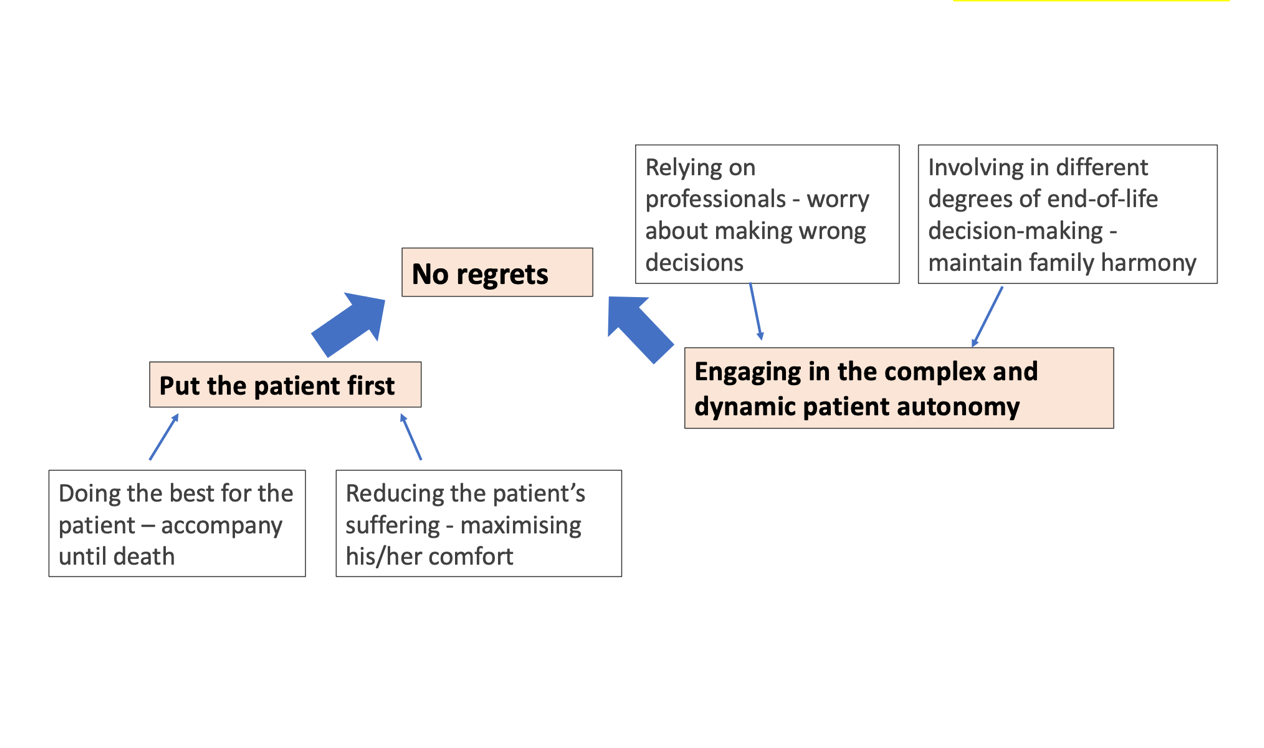


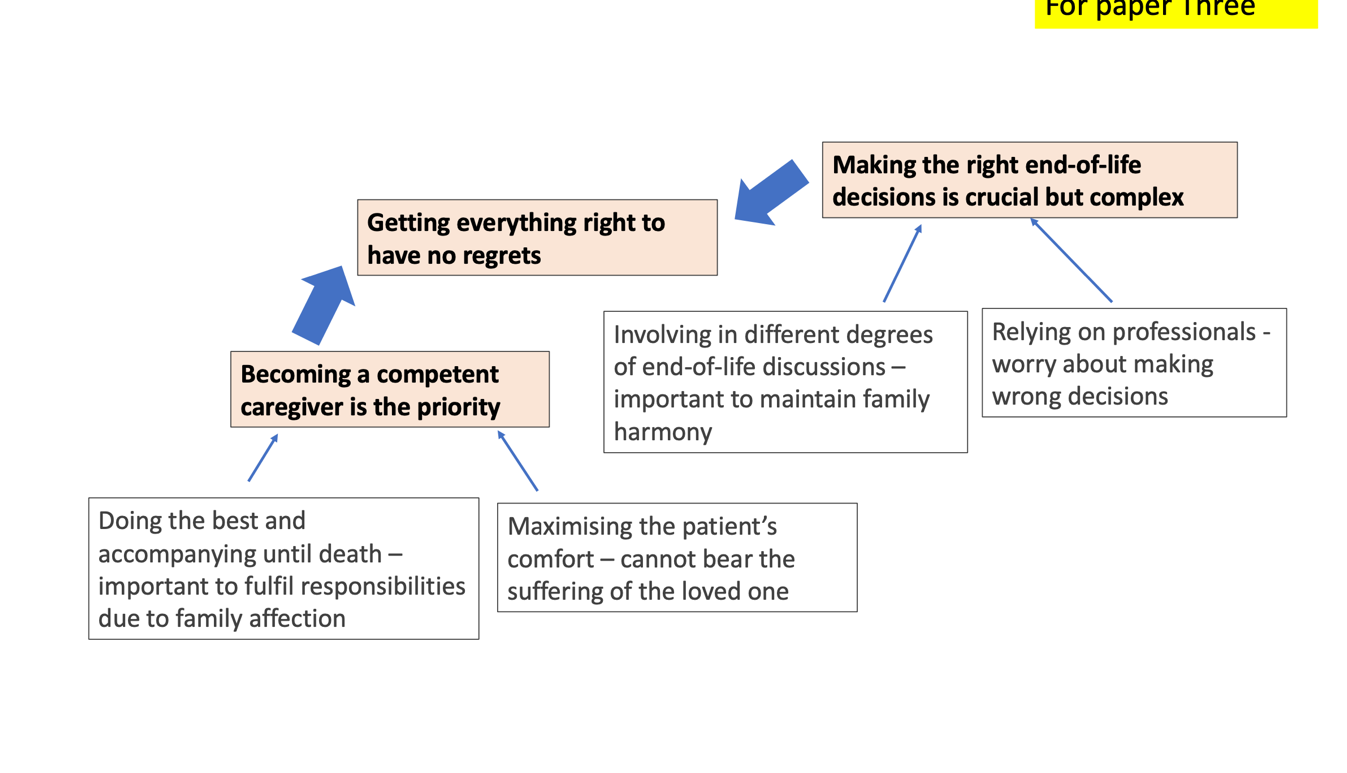


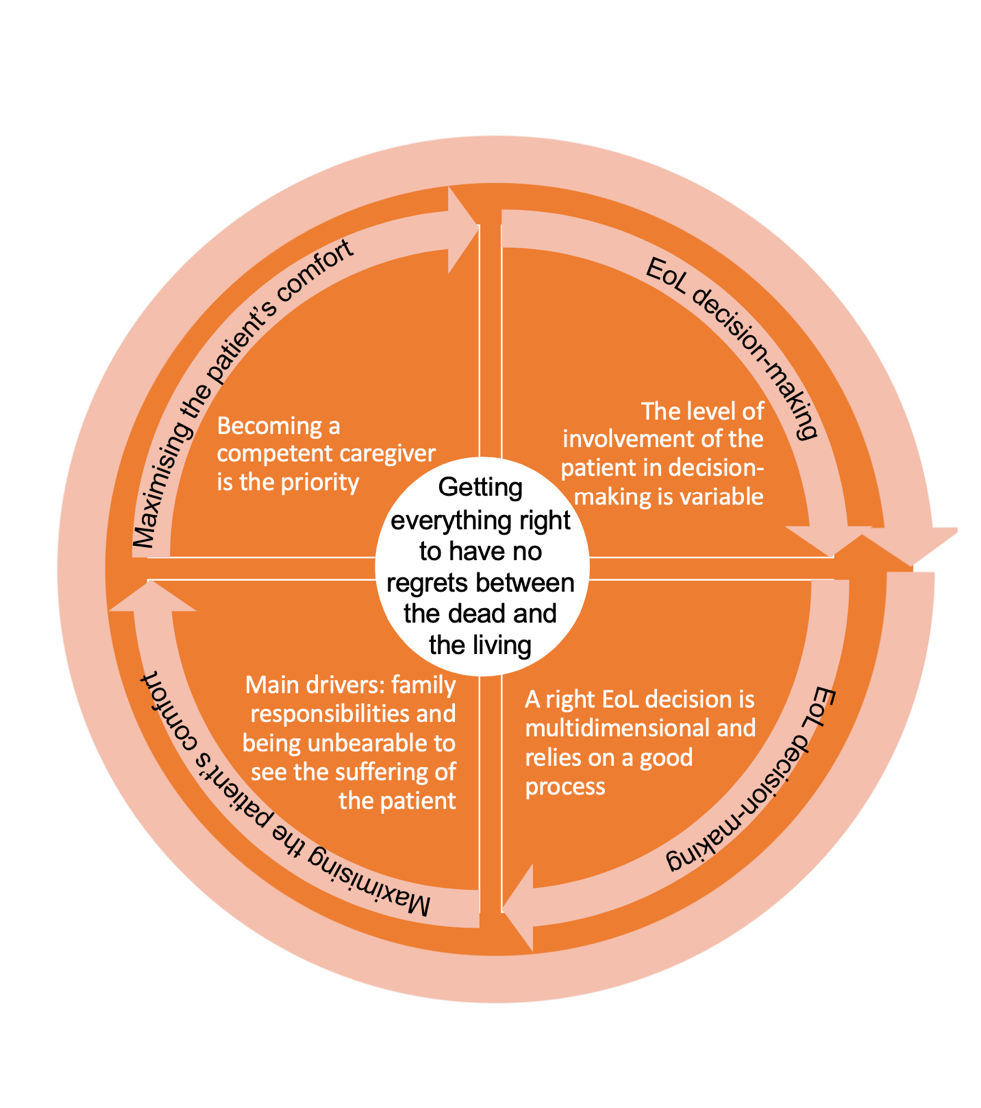

Supplement: sj-docx-1-pmj-10.1177_02692163251316677 – Supplemental material for ‘Regrets become a lasting source of pain’: A qualitative study on family caregivers’ experiences leading up to a relative’s death [file sj-docx-1-pmj-10.1177_02692163251316677.docx]
